# 间充质干细胞在移植物抗宿主病中的研究进展

**DOI:** 10.3760/cma.j.cn121090-20250925-00444

**Published:** 2026-06

**Authors:** 文浩 王, 晓磊 裴, 洁雅 骆, 尔烈 姜

**Affiliations:** 1 天津医科大学，细胞生态海河实验室，天津 300070 Haihe Laboratory of Cell Ecosystem, Tianjin Medical University, Tianjin 300070, China; 2 中国医学科学院血液病医院（中国医学科学院血液学研究所），血液与健康全国重点实验室，国家血液系统疾病临床医学研究中心，细胞生态海河实验室，天津 300020 State Key Laboratory of Experimental Hematology, National Clinical Research Center for Blood Diseases, Haihe Laboratory of Cell Ecosystem, Institute of Hematology & Blood Diseases Hospital, Chinese Academy of Medical Sciences & Peking Union Medical College, Tianjin 300020, China; 3 天津医学健康研究院，天津 301600 Tianjin Institutes of Health Science, Tianjin 301600, China

## Abstract

移植物抗宿主病（GVHD）是异基因造血干细胞移植后的严重并发症，尤其是对于激素难治或经多线治疗失败的患者，目前仍缺乏有效的治疗手段。间充质干细胞（MSC）因具有低免疫原性以及免疫调节、抗炎和组织修复等特性，在GVHD防治中展现出良好的应用潜力。目前，包括我国在内的多个国家已批准MSC产品用于急性GVHD（aGVHD）的治疗。尽管该疗法耐受性较好，但仍面临疗效个体差异较大、潜在安全风险以及生产与质量控制标准不统一等挑战。本文综述了MSC用于GVHD干预的最新临床研究进展，并探讨该疗法的应用前景与现存局限，为推动其规范化、精准化应用提供参考。

## Abstract (en)

Graft-versus-host disease（GVHD）remains a severe complication after allogeneic hematopoietic stem cell transplantation, and effective treatment options remain limited, particularly for patients with steroid-refractory GVHD or failure of multiple lines of therapy. Mesenchymal stem cell（MSC）therapy has shown promise for the prevention and treatment of GVHD because of the low immunogenicity and immunomodulatory, anti-inflammatory, and tissue-reparative properties of MSC. To date, MSC-based products have been approved for the treatment of acute GVHD（aGVHD）in several countries, including China. Although MSC therapy is generally well tolerated, its broader clinical application remains challenged by substantial interindividual variability in therapeutic response, potential safety concerns, and the lack of harmonized standards for manufacturing and quality control. This review systematically summarizes recent clinical research on MSC-based interventions for GVHD and critically discusses their therapeutic prospects and current limitations, with the aim of informing standardized and precision-oriented clinical application of MSC therapy.

移植物抗宿主病（graft-versus-host disease，GVHD）是异基因造血干细胞移植（allogeneic hematopoietic stem cell transplantation, allo-HSCT）后的主要并发症之一，显著增加患者的非复发死亡率（non-relapse mortality, NRM）。近年来，随着GVHD预防和治疗策略的不断更新，相关研究取得了一定进展，但临床实践中仍面临巨大挑战[1]。根据发病时间和临床表现的差异，GVHD可分为急性移植物抗宿主病（acute graft-versus-host disease, aGVHD）和慢性移植物抗宿主病（chronic graft-versus-host disease, cGVHD）[2]。糖皮质激素是aGVHD和cGVHD的一线标准治疗，但在实际治疗中，35％～50％的aGVHD患者对激素不敏感，其2年总生存（overall survival, OS）率低于20％[3]。中重度cGVHD患者往往需要长期依靠激素维持疗效，生活质量显著下降[4]。尽管多种二线治疗显示出一定疗效，但患者间的治疗反应差异显著，最佳二线治疗方案仍有待明确。

间充质干细胞（mesenchymal stem cell, MSC）作为多能干细胞，主要来源于骨髓、脂肪组织及脐带，具有多向分化潜能、免疫调节作用及组织修复能力[5]。MSC具有低免疫原性、多靶点作用等特点，使其在激素难治性GVHD（steroid-refractory GVHD, SR-GVHD）的治疗中展现出独特优势，有望成为一种更安全、有效的治疗选择[6]。本文旨在综述近年来MSC在GVHD防治中的临床研究进展，重点评估其临床疗效与安全性，并探讨相关的潜在作用机制，以期为未来的临床应用和基础研究提供参考。

一、MSC在GVHD预防和治疗中的临床应用

2004年，Le Blanc团队[7]首次报道了MSC成功治疗儿童重度aGVHD的案例，标志着MSC在GVHD治疗中迈进了临床应用阶段。此后二十年间，MSC在GVHD中的应用探索不断深入，其临床策略已从最初的挽救性治疗扩展至一线联合乃至预防性应用。近年来，一系列重要的临床研究结果相继发表，进一步揭示了MSC的疗效、安全性与面临的挑战。

（一）GVHD的预防

随着对GVHD发病机制认识的不断加深，其免疫预防策略已不再局限于传统的“钙调磷酸酶抑制剂联合抗代谢药物”模式，而是逐步探索更为灵活的组合预防方案[8]。鉴于MSC兼具免疫调节与辅助造血重建的双重潜能，其在allo-HSCT后的预防性应用受到广泛关注[9]。

MSC可通过旁分泌多种免疫调节因子，促进调节性T细胞（regulatory T cell, Treg）的诱导与扩增，同时抑制效应T细胞的活化和增殖，并减少TNF-α、IFN-γ等促炎性细胞因子的释放，从而在移植早期塑造有利于免疫耐受建立的微环境[10]–[11]。此外，MSC还可通过抑制树突状细胞的分化与成熟，降低其抗原呈递能力和T细胞刺激功能，从而在多个层面协同减弱供受者间的免疫激活反应[12]–[13]。

近年来，其预防效果已在多项研究中得到验证。在Wang等[14]开展的一项多中心回顾性研究中，17例原发性骨髓纤维化患者在接受allo-HSCT的同时输注了体外扩增的MSC。结果显示，2年累计cGVHD发病率为63％（95％ *CI*: 26％～81％），但中重度cGVHD发生率仅为17％（95％ *CI*: 0％～42％）。尽管样本量较少，该研究仍提示了MSC的潜在价值。Lombardo等[15]在HLA不合无关供者移植研究中发现，共同输注骨髓来源MSC（bone marrow-derived mesenchymal stem cell, BM-MSC）可显著降低Ⅱ～Ⅳ级aGVHD发生率（*HR*＝0.332，95％ *CI*: 0.124～0.890，*P*＝0.028），但在预防cGVHD方面未见显著差异。相比之下，Huang等[16]的多中心随机临床试验显示，单倍体相合移植后早期序贯输注脐带来源MSC（umbilical cord-derived mesenchymal stem cell, UC-MSC），MSC组中重度cGVHD的2年累积发生率为5.4％，显著低于对照组的17.4％（*P*＝0.03），且Ⅱ～Ⅳ级aGVHD发生率也明显下降（*P*＝0.01），并显著提升了患者的无GVHD无复发生存（graft-versus-host disease-free, relapse-free survival, GRFS）（*P*＝0.04）。近期一项涵盖8项随机对照试验（randomized controlled trial, RCT）的Meta分析提示MSC在降低GVHD风险及提升OS率方面具有一定安全性与有效性；亚组分析进一步指出，UC-MSC在预防效果上优于BM-MSC，这可能与其更强的免疫调节能力及更长的体内存活时间有关[17]。

（二）aGVHD的治疗

aGVHD是MSC在HSCT领域应用最为广泛且研究最深入的适应证之一，尤其是SR-aGVHD。2024年12月，美国食品药品监督管理局批准了Remestemcel-L（骨髓来源MSC）用于治疗儿童SR-aGVHD。Kurtzberg等[18]针对Remestemcel-L的儿童单臂Ⅲ期研究（*n*＝54）显示，治疗第28天客观缓解率（overall response rate, ORR）达70.4％，显著优于历史对照组（45％，*P*<0.001），且第28天的应答状态显著预测了第100天的生存结局。另一项Kebriaei等[19]开展的Ⅲ期RCT（*n*＝260）评估了Remestemcel-L联合二线治疗效果，发现肝脏受累患者的持续完全缓解（durable complete response，DCR）率更高（29％对5％，*P*＝0.047），这可能与抑制肝脏TNF-α和IFN-γ分泌有关。与之相反，Niu等[20]的研究提示肝脏受累是成人重症SR-aGVHD预后不良的因素，接受UC-MSC治疗的肝脏受累患者疗效较差。这种差异可能与研究使用的不同MSC来源、患者亚组差异以及治疗方案有关。

随后，艾米迈托赛注射液（人UC-MSC）于2025年1月在我国获批上市，用于治疗14岁以上消化道受累为主的SR-aGVHD患者，为国内患者提供了新的治疗选择。这一获批主要基于MSC在肠道受累型aGVHD中展现的治疗潜力。在一项Ⅱ期多中心随机、双盲、安慰剂对照试验（*n*＝78）[21]中，尽管MSC组与对照组治疗后第28天ORR差异无统计学意义（60％对50％，*P*＝0.375），但在肠道受累患者中，MSC组治疗后第28天ORR显著高于对照组（66.7％对33.3％，*P*＝0.031）。

MSC并非仅在儿童中有效，在成人重症及多器官受累人群中同样可见疗效。Zhao等[22]的Ⅲ期RCT（*n*＝203）及另一项多中心RCT（NCT04738981, *n*＝130）[23]均显示，MSC联合CD25单抗治疗SR-aGVHD的效果显著优于单药，且在多器官受累的重症患者中优势更为明显。这一协同效应的机制可能为CD25单抗通过阻断IL-2Rα，抑制活化T细胞增殖，但间接削弱Treg的扩增，造成免疫耐受重建障碍[24]，而MSC通过上调HO-1与TGF-β，从而诱导Treg增殖并抑制Th17细胞[25]–[26]，对上述免疫失衡进行调节。

针对芦可替尼难治性aGVHD（ruxolitinib-refractory acute graft-versus-host disease, RR-aGVHD）人群，BM-MSC同样展现出较好的治疗效果。Bonig等[27]回顾性分析156例应用BM-MSC的RR-aGVHD患者（含33例儿童）发现，整个队列治疗后28 d ORR为49％，其中儿童高达64％；6个月OS率成人为47％、儿童为59％，显著优于历史队列的20％，提示MSC在高度难治人群中仍具有临床价值。相关基础研究进一步表明，芦可替尼可通过JAK2/STAT1通路直接修复MSC功能，进而改善aGVHD所致的造血功能障碍[28]。Ryan等[29]进一步证实，芦可替尼阻断IFN-γ诱导的骨髓MSC中STAT1磷酸化，抑制HLA-DR、CX3CL1、CCL2等分子，并逆转IFN-γ对MSC的增殖抑制。这些发现为二者联合应用提供了理论依据。

MSC在经典aGVHD与晚发aGVHD中的疗效可能存在差异。Keklik等[30]对76例使用脂肪或脐带来源MSC的重症aGVHD患者进行分析发现，MSC在晚发aGVHD患者中的疗效优于经典aGVHD患者。其完全缓解（complete response, CR）率（23.3％对10.9％）、部分缓解（partial response, PR）率（36.7％对23.9％）、2年OS率（59％对28％）以及NRM（40％对71％）均显著改善，这可能与晚发aGVHD免疫病理特征有关，其炎症环境相对更易被MSC调节，从而使MSC的免疫抑制和组织修复作用更有效。

综上，MSC在aGVHD的治疗中显示出较好的疗效及耐受性，为多器官受累及多线治疗失败患者提供一种新的治疗选择。然而，至今尚缺乏随机对照试验对上述不同来源MSC的治疗特性进行直接比较，未来仍需进一步明确不同临床亚型中最优的细胞来源及给药策略。

（三）cGVHD的治疗

尽管MSC在aGVHD治疗中的应用研究较为成熟，但其在cGVHD中的临床研究较少，现有证据主要来源于小样本研究。Boberg等[31]报道，在接受MSC重复输注的11例cGVHD患者中，有6例（54.5％）获得临床应答，其中2例能够停用全身免疫抑制治疗。进一步分析发现，应答者具有较高的初始T/B细胞水平、较高比例的CD31^+^初始CD4^+^ T细胞，并伴有CXCL9、CXCL10等趋化因子的动态变化。在另一项研究中[32]，10例激素难治性cGVHD患者接受4次BM-MSC输注后均于8周时达到PR，1年OS率高达80％，且炎症标志物ST2、CXCL10和SPP1水平显著下降，进一步支持了MSC改善炎症微环境的作用。在更大的临床队列中，MSC同样表现出稳定的疗效。西班牙的一项多中心回顾性研究[33]共纳入62例患者，其中53例接受了脂肪组织来源MSC（adipose tissue-derived mesenchymal stem cells, AT-MSC）治疗，8例接受BM-MSC治疗；结果显示，aGVHD患者的ORR为58.7％，cGVHD患者的ORR为62.5％。即便是对于多药难治GVHD（multidrug-refractory GVHD, MDR-GVHD）患者，MSC也表现出一定效果[34]：MDR-aGVHD患者ORR为57.1％（8/14），MDR-cGVHD患者为42.9％（3/7）。

综上，MSC在cGVHD治疗中同样显示出一定的治疗潜力，并在部分激素难治或多药治疗失败患者中观察到一定疗效，未来有必要通过进一步研究明确适宜的患者人群、治疗时机及疗效预测指标。

二、不同来源MSC对比

MSC的来源较为广泛，目前主要包括骨髓、脐带、脂肪组织和其他新兴来源。但这些不同来源MSC在免疫表型、扩增能力、免疫调节能力等方面并非完全一致[35]，这些差异可能进一步影响其临床表现与适用场景。

从生物学特性来看，BM-MSC最早被分离鉴定，具有较强的免疫调节能力和维持造血功能的能力，有丰富的基础和临床数据[36]–[37]。其获取过程具有一定侵入性，且供者年龄可显著影响细胞质量，这些因素限制了其进一步推广[38]。尽管如此，BM-MSC仍具有较强的免疫抑制活性，体外比较研究显示，在未经IFN-γ和TNF-α预激的条件下，BM-MSC与AT-MSC抑制淋巴细胞增殖的能力均强于UC-MSC[39]。

相比之下，UC-MSC凭借无创获取、来源充足、伦理争议较少以及低免疫原性受到广泛关注[40]；其通常来源于分娩后的脐带组织，不会对供者造成额外伤害，并且表现出较高的增殖潜能以及相对稳定的生物学特性[41]。华通胶来源间充质干细胞（Wharton's jelly-derived mesenchymal stem cells, WJ-MSC）是一种分离自脐带华通胶的MSC，研究显示其较多数成体组织来源MSC具有更强的增殖能力和免疫抑制活性，并可能具有更大的治疗潜力[42]–[43]。

AT-MSC同样因获取相对简便、来源丰富而受到关注，具有较高的增殖潜能和抗炎特性[44]，已有研究发现，与BM-MSC及UC-MSC相比，AT-MSC在造血支持能力、抗补体裂解能力及体内存活率等方面显示出一定优势[45]–[46]，可能成为GVHD治疗中另一种有价值的选择。

诱导多能干细胞衍生的间充质干细胞（induced pluripotent stem cell-derived mesenchymal stem cells, iMSC）由人诱导多能干细胞经定向分化获得。与成人MSC相比，iMSC具有“几乎无限”的扩增能力和更好的遗传一致性[47]，长达2年的随访研究为其临床应用可行性提供了初步支持[48]。

除上述来源外，部分新兴组织来源MSC也受到关注。胎盘来源的间充质干细胞（placenta-derived mesenchymal stem cells, P-MSC）是从胎盘组织多个部位分离获得的，其收集过程相对无创、来源丰富，同样具有良好的免疫调节能力[49]。齿龈来源的间充质干细胞（gingiva-derived MSC, GMSC）来自口腔齿龈组织，具有易于获取、微创分离的优势；与BM-MSC或AT-MSC相比，GMSC可能具有更强的保护效能和扩增能力，同样具备治疗GVHD的潜力[50]。

在安全性层面，目前的循证医学证据为MSC的临床应用提供了一定支持。纳入129项研究、涉及超过6 000例患者的3项Meta分析一致表明[51]–[53]，相比传统疗法，MSC并未显著增加严重感染、血栓事件、恶性肿瘤或死亡风险，不良反应多为输注时一过性发热以及局部反应，且多呈自限性；在长达5年的随访中，亦未观察到严重的迟发性不良反应。然而，这种整体上的安全性评价并不意味着各来源MSC的风险特征完全一致。多项研究提示，UC-MSC相较于其他来源具有更高的血栓风险[39],[54]–[55]。此外，当前的研究主要基于总体人群，针对老年患者、合并肝肾功能不全患者、既往有血栓病史患者等临床常见特殊人群的数据仍然有限，其安全性及疗效是否与常规人群一致，尚有待进一步研究加以验证。

不同来源MSC的对比详见表1。

**表1:** 不同来源间充质干细胞（MSC）生物学特征、临床应用及安全性比较

来源	关键特征	临床应用	安全性	局限性	参考文献
骨髓来源MSC	造血微环境支持能力强	用于GVHD的二线或挽救治疗研究，也可用于儿童	输注相关不良反应报道较少，整体耐受性良好	采集过程侵入性较高，细胞产量有限，供者间差异明显，受供者衰老影响	[36]–[38]
脐带来源MSC	增殖能力强，低免疫原性	在aGVHD中应用广泛，对胃肠道受累患者可能具有较好反应	总体安全性良好，部分研究提示血栓风险相对较高	血栓风险，供者异质性	[39]–[41],[54]–[55]
脂肪组织来源MSC	易于获取，细胞产量高	多为小样本探索	现有小样本研究中尚未见明显严重不良反应	治疗GVHD证据有限	[44]–[46]
诱导多能干细胞衍生MSC	“无限扩增”，遗传一致性高	处于早期临床研究阶段	临床安全性及长期风险尚待进一步验证	潜在致瘤风险	[47]–[48]
新兴组织来源MSC^a^	易于获取，高增殖能力	处于早期临床研究阶段	临床安全性数据有限，仍需进一步验证	临床数据有限	[49]–[50]

**注** GVHD：移植物抗宿主病；aGVHD：急性移植物抗宿主病。^a^新兴组织包括胎盘、齿龈等

三、MSC治疗GVHD的挑战与展望

由于具有独特的生物学特性、来源多样且伦理可接受性较高，MSC近年来逐步成为研究热点，已有多个国家批准其用于GVHD治疗（表2）。然而，目前临床实践中仍存在疗效不稳定、缺乏统一标准等问题，限制了其进一步规范化应用。针对上述问题，后续研究可从以下三个层面推进。

**表2:** 已批准用于治疗移植物抗宿主病的间充质干细胞产品概览

名称	适应证	批准日期	获批地区	来源
艾米迈托赛注射液	用于治疗14岁以上消化道受累为主的SR-aGVHD	2025	中国	脐带
Ryoncil（Remestemcel-L）	适用于治疗2个月及以上儿童SR-aGVHD	2024	美国	骨髓
TEMCELL	用于治疗aGVHD	2014	日本	骨髓
Prochymal	儿童aGVHD	2012	加拿大	骨髓
Prochymal	儿童aGVHD	2012	新西兰	骨髓

**注** SR-GVHD：激素难治性移植物抗宿主病；aGVHD：急性移植物抗宿主病

（一）MSC疗效异质性的影响因素及分层应对策略

不同来源MSC在增殖潜能、免疫调节能力及对炎症微环境的响应性上存在显著差异，是导致临床疗效不一致的重要原因之一。研究显示，MSC供者年龄可能影响其体内治疗效能：与年龄较大的供者相比，年轻供者来源MSC治疗的SR-aGVHD患者具有更高的1年总生存率[56]。此外，患者淋巴细胞计数、血小板计数及总胆红素等指标与治疗反应密切相关，为探索基于生物标志物的患者分层提供了依据[57]。

（二）MSC预处理策略的优化方向

为提高MSC在体内的存活、归巢及免疫调节能力，低氧、活性分子及基因修饰等多种预处理策略已成为提升疗效的重要路径[58]–[59]。近期研究发现，经IFN-γ预处理的MSC在GVHD动物模型中表现出更显著的治疗优势[60]；AKT1修饰可增强MSC的免疫调节能力，并改善GVHD相关免疫反应[61]。此外，代谢重编程[62]和表观遗传重编程[63]等策略有望提高不同个体来源MSC的一致性。

（三）制备、保存及质量控制的标准化路径

在临床应用中，MSC通常需要冷冻保存，而冷冻保存及复苏过程可能损害其代谢活性[64]。因此，优化冷冻保存与复苏方案，减少储存、运输和复苏等关键环节的温度波动，对MSC制剂的临床转化至关重要[65]。在生产环节，应严格遵循药品生产质量管理规范，通过标准化细胞来源、优化培养基选择、控制传代次数并加强环境监控，以提升产品的批次稳定性[66]。此外，应积极推广自动化、封闭式生产系统的应用。通过在线监测pH值、代谢产物浓度及细胞密度等关键工艺参数，可实现生产过程的精准控制，并最大程度减少人为因素干扰[67]。基于标准化制造和质量控制体系制备的MSC制剂，更有可能在不同中心获得相对一致的临床疗效，推动MSC的规范化应用。

四、结语

综上所述，MSC凭借其免疫调节、抗炎及组织修复潜能，已在GVHD的预防与治疗中展现出良好的应用前景。随着机制研究的深入、制备工艺的优化及质量控制体系的完善，MSC有望在GVHD的精准干预与个体化治疗中发挥更加重要的作用。

## References

[1] Penack O, Marchetti M, Aljurf M (2024). Prophylaxis and management of graft-versus-host disease after stem-cell transplantation for haematological malignancies: updated consensus recommendations of the European Society for Blood and Marrow Transplantation[J]. Lancet Haematol.

[2] Filipovich AH, Weisdorf D, Pavletic S (2005). National Institutes of Health consensus development project on criteria for clinical trials in chronic graft-versus-host disease: I. Diagnosis and staging working group report[J]. Biol Blood Marrow Transplant.

[3] Westin JR, Saliba RM, De Lima M (2011). Steroid-Refractory Acute GVHD: Predictors and Outcomes[J]. Adv Hematol.

[4] Wolff D, Fatobene G, Rocha V (2021). Steroid-refractory chronic graft-versus-host disease: treatment options and patient management[J]. Bone Marrow Transplant.

[5] Zhou T, Yuan Z, Weng J (2021). Challenges and advances in clinical applications of mesenchymal stromal cells[J]. J Hematol Oncol.

[6] Kadri N, Amu S, Iacobaeus E (2023). Current perspectives on mesenchymal stromal cell therapy for graft versus host disease[J]. Cell Mol Immunol.

[7] Le Blanc K, Rasmusson I, Sundberg B (2004). Treatment of severe acute graft-versus-host disease with third party haploidentical mesenchymal stem cells[J]. Lancet.

[8] 中华医学会血液学分会干细胞应用学组 (2024). 异基因造血干细胞移植急性移植物抗宿主病诊断与治疗中国专家共识(2024年版)[J]. 中华血液学杂志.

[9] Deng M, Zhang X, Zhang Y (2025). The roles and clinical applications of mesenchymal stem cells and their exosomes in hematologic diseases[J]. Stem Cell Res Ther.

[10] Aggarwal S, Pittenger MF (2005). Human mesenchymal stem cells modulate allogeneic immune cell responses[J]. Blood.

[11] Azevedo RI, Minskaia E, Fernandes-Platzgummer A (2020). Mesenchymal stromal cells induce regulatory T cells via epigenetic conversion of human conventional CD4 T cells in vitro[J]. Stem Cells.

[12] Jiang XX, Zhang Y, Liu B (2005). Human mesenchymal stem cells inhibit differentiation and function of monocyte-derived dendritic cells[J]. Blood.

[13] Djouad F, Charbonnier LM, Bouffi C (2007). Mesenchymal stem cells inhibit the differentiation of dendritic cells through an interleukin-6-dependent mechanism[J]. Stem Cells.

[14] Wang Q, Xu N, Wang Y (2022). Allogeneic Stem Cell Transplantation Combined With Transfusion of Mesenchymal Stem Cells in Primary Myelofibrosis: A Multicenter Retrospective Study[J]. Front Oncol.

[15] Lombardo G, Lechanteur C, Briquet A (2024). Co-infusion of mesenchymal stromal cells to prevent GVHD after allogeneic hematopoietic cell transplantation from HLA-mismatched unrelated donors after reduced-intensity conditioning: a double-blind randomized study and literature review[J]. Stem Cell Res Ther.

[16] Huang R, Chen T, Wang S (2024). Mesenchymal Stem Cells for Prophylaxis of Chronic Graft-vs-Host Disease After Haploidentical Hematopoietic Stem Cell Transplant: An Open-Label Randomized Clinical Trial[J]. JAMA Oncol.

[17] Rameshbabu S, Jayaraman Kannan H, Moondra M (2025). Mesenchymal stromal cells for the prophylaxis of graft-versus-host disease after hematopoietic stem cell transplantation: a meta-analysis of randomized controlled trials[J]. Expert Rev Hematol.

[18] Kurtzberg J, Abdel-Azim H, Carpenter P (2020). A Phase 3, Single-Arm, Prospective Study of Remestemcel-L, Ex Vivo Culture-Expanded Adult Human Mesenchymal Stromal Cells for the Treatment of Pediatric Patients Who Failed to Respond to Steroid Treatment for Acute Graft-versus-Host Disease[J]. Biol Blood Marrow Transplant.

[19] Kebriaei P, Hayes J, Daly A (2020). A Phase 3 Randomized Study of Remestemcel-L versus Placebo Added to Second-Line Therapy in Patients with Steroid-Refractory Acute Graft-versus-Host Disease[J]. Biol Blood Marrow Transplant.

[20] Niu JW, Li Y, Xu C (2024). Human umbilical cord-derived mesenchymal stromal cells for the treatment of steroid refractory grades III-IV acute graft-versus-host disease with long-term follow-up[J]. Front Immunol.

[21] Jiang E, Qian K, Wang L (2024). Efficacy and safety of human umbilical cord-derived mesenchymal stem cells versus placebo added to second-line therapy in patients with steroid-refractory acute graft-versus-host disease: a multicentre, randomized, double-blind, phase 2 trial[J]. BMC Med.

[22] Zhao K, Lin R, Fan Z (2022). Mesenchymal stromal cells plus basiliximab, calcineurin inhibitor as treatment of steroid-resistant acute graft-versus-host disease: a multicenter, randomized, phase 3, open-label trial[J]. J Hematol Oncol.

[23] Fu H, Sun X, Lin R (2024). Mesenchymal stromal cells plus basiliximab improve the response of steroid-refractory acute graft-versus-host disease as a second-line therapy: a multicentre, randomized, controlled trial[J]. BMC Med.

[24] Chakupurakal G, García-Márquez MA, Shimabukuro-Vornhagen A (2016). Immunological effects in patients with steroid-refractory graft-versus-host disease following treatment with basiliximab, a CD25 monoclonal antibody[J]. Eur J Haematol.

[25] Negi N, Griffin MD (2020). Effects of mesenchymal stromal cells on regulatory T cells: Current understanding and clinical relevance[J]. Stem Cells.

[26] Terraza-Aguirre C, Campos-Mora M, Elizondo-Vega R (2020). Mechanisms behind the Immunoregulatory Dialogue between Mesenchymal Stem Cells and Th17 Cells[J]. Cells.

[27] Bonig H, Verbeek M, Herhaus P (2023). Real-world data suggest effectiveness of the allogeneic mesenchymal stromal cells preparation MSC-FFM in ruxolitinib-refractory acute graft-versus-host disease[J]. J Transl Med.

[28] Lin Y, Gu Q, Lu S (2023). Ruxolitinib improves hematopoietic regeneration by restoring mesenchymal stromal cell function in acute graft-versus-host disease[J]. J Clin Invest.

[29] Ryan MM, Patel M, Hogan K (2021). Ruxolitinib Inhibits IFNγ Licensing of Human Bone Marrow Derived Mesenchymal Stromal Cells[J]. Transplant Cell Ther.

[30] Keklik M, Deveci B, Celik S (2023). Safety and efficacy of mesenchymal stromal cell therapy for multi-drug-resistant acute and late-acute graft-versus-host disease following allogeneic hematopoietic stem cell transplantation[J]. Ann Hematol.

[31] Boberg E, von Bahr L, Afram G (2020). Treatment of chronic GvHD with mesenchymal stromal cells induces durable responses: A phase II study[J]. Stem Cells Transl Med.

[32] Kim N, Min GJ, Im KI (2024). Repeated Infusions of Bone-Marrow-Derived Mesenchymal Stem Cells over 8 Weeks for Steroid-Refractory Chronic Graft-versus-Host Disease: A Prospective, Phase I/II Clinical Study[J]. Int J Mol Sci.

[33] Macías-Sánchez MDM, Morata-Tarifa C, Cuende N (2022). Mesenchymal Stromal Cells for Treating Steroid-Resistant Acute and Chronic Graft Versus Host Disease: A Multicenter Compassionate Use Experience[J]. Stem Cells Transl Med.

[34] Shen MZ, Liu XX, Qiu ZY (2022). Efficacy and safety of mesenchymal stem cells treatment for multidrug-resistant graft-versus-host disease after haploidentical allogeneic hematopoietic stem cell transplantation[J]. Ther Adv Hematol.

[35] Heo JS, Choi Y, Kim HS (2016). Comparison of molecular profiles of human mesenchymal stem cells derived from bone marrow, umbilical cord blood, placenta and adipose tissue[J]. Int J Mol Med.

[36] Müller L, Tunger A, Wobus M (2021). Immunomodulatory Properties of Mesenchymal Stromal Cells: An Update[J]. Front Cell Dev Biol.

[37] Bačenková D, Trebuňová M, Dosedla E (2025). Interactions of Hematopoietic and Associated Mesenchymal Stem Cell Populations in the Bone Marrow Microenvironment, In Vivo and In Vitro Model[J]. Int J Mol Sci.

[38] Wu LW, Wang YL, Christensen JM (2014). Donor age negatively affects the immunoregulatory properties of both adipose and bone marrow derived mesenchymal stem cells[J]. Transpl Immunol.

[39] Grégoire C, Ritacco C, Hannon M (2019). Comparison of Mesenchymal Stromal Cells From Different Origins for the Treatment of Graft-vs.-Host-Disease in a Humanized Mouse Model[J]. Front Immunol.

[40] Jin HJ, Bae YK, Kim M (2013). Comparative analysis of human mesenchymal stem cells from bone marrow, adipose tissue, and umbilical cord blood as sources of cell therapy[J]. Int J Mol Sci.

[41] Patel AA, Mohamed AH, Rizaev J (2024). Application of mesenchymal stem cells derived from the umbilical cord or Wharton's jelly and their extracellular vesicles in the treatment of various diseases[J]. Tissue Cell.

[42] Marino L, Castaldi MA, Rosamilio R (2019). Mesenchymal Stem Cells from the Wharton's Jelly of the Human Umbilical Cord: Biological Properties and Therapeutic Potential[J]. Int J Stem Cells.

[43] Kim DW, Staples M, Shinozuka K (2013). Wharton's jelly-derived mesenchymal stem cells: phenotypic characterization and optimizing their therapeutic potential for clinical applications[J]. Int J Mol Sci.

[44] Wang L, Jiang X, Zhao F (2025). A review of adipose-derived mesenchymal stem cells' impacts and challenges: metabolic regulation, tumor modulation, immunomodulation, regenerative medicine and genetic engineering therapies[J]. Front Endocrinol (Lausanne).

[45] Wu SCM, Zhu M, Chik SCC (2023). Adipose tissue-derived human mesenchymal stromal cells can better suppress complement lysis, engraft and inhibit acute graft-versus-host disease in mice[J]. Stem Cell Res Ther.

[46] Jiang M, Bi X, Duan X (2020). Adipose tissue-derived stem cells modulate immune function in vivo and promote long-term hematopoiesis in vitro using the aGVHD model[J]. Exp Ther Med.

[47] Wu Z, Su Y, Li J (2024). Induced pluripotent stem cell-derived mesenchymal stem cells: whether they can become new stars of cell therapy[J]. Stem Cell Res Ther.

[48] Kelly K, Bloor AJC, Griffin JE (2024). Two-year safety outcomes of iPS cell-derived mesenchymal stromal cells in acute steroid-resistant graft-versus-host disease[J]. Nat Med.

[49] Talwadekar MD, Kale VP, Limaye LS (2015). Placenta-derived mesenchymal stem cells possess better immunoregulatory properties compared to their cord-derived counterparts-a paired sample study[J]. Sci Rep.

[50] Ni X, Xia Y, Zhou S (2019). Reduction in murine acute GVHD severity by human gingival tissue-derived mesenchymal stem cells via the CD39 pathways[J]. Cell Death Dis.

[51] Thompson M, Mei SHJ, Wolfe D (2020). Cell therapy with intravascular administration of mesenchymal stromal cells continues to appear safe: An updated systematic review and meta-analysis[J]. EClinicalMedicine.

[52] Li T, Luo C, Zhang J (2021). Efficacy and safety of mesenchymal stem cells co-infusion in allogeneic hematopoietic stem cell transplantation: a systematic review and meta-analysis[J]. Stem Cell Res Ther.

[53] Wang Y, Yi H, Song Y (2021). The safety of MSC therapy over the past 15 years: a meta-analysis[J]. Stem Cell Res Ther.

[54] Moll G, Ankrum JA, Olson SD (2022). Improved MSC Minimal Criteria to Maximize Patient Safety: A Call to Embrace Tissue Factor and Hemocompatibility Assessment of MSC Products[J]. Stem Cells Transl Med.

[55] Hao X, Zhu H, Qin C (2024). Study on Preclinical Safety and Toxic Mechanism of Human Umbilical Cord Mesenchymal Stem Cells in F344RG Rats[J]. Stem Cell Rev Rep.

[56] van der Wagen LE, Miranda-Bedate A, Janssen A (2020). Efficacy of MSC for steroid-refractory acute GVHD associates with MSC donor age and a defined molecular profile[J]. Bone Marrow Transplant.

[57] Hinden L, Avner M, Stepensky P (2019). Lymphocyte counts may predict a good response to mesenchymal stromal cells therapy in graft versus host disease patients[J]. PLoS One.

[58] Ocansey DKW, Pei B, Yan Y (2020). Improved therapeutics of modified mesenchymal stem cells: an update[J]. J Transl Med.

[59] Moeinabadi-Bidgoli K, Mazloomnejad R, Beheshti Maal A (2023). Genetic modification and preconditioning strategies to enhance functionality of mesenchymal stromal cells: a clinical perspective[J]. Expert Opin Biol Ther.

[60] Pochon C, Perouf R, Notarantonio AB (2025). Optimized GMP-grade Wharton's jelly's mesenchymal stromal cells manufacturing and administration protocol for Graft versus Host disease prevention[J]. Curr Res Transl Med.

[61] Zhou L, Liu S, Wang Z (2019). Bone Marrow-Derived Mesenchymal Stem Cells Modified with Akt1 Ameliorates Acute Liver GVHD[J]. Biol Proced Online.

[62] Yuan X, Logan TM, Ma T (2019). Metabolism in Human Mesenchymal Stromal Cells: A Missing Link Between hMSC Biomanufacturing and Therapy?[J]. Front Immunol.

[63] Walewska A, Janucik A, Tynecka M (2023). Mesenchymal stem cells under epigenetic control - the role of epigenetic machinery in fate decision and functional properties[J]. Cell Death Dis.

[64] Yin JQ, Zhu J, Ankrum JA (2019). Manufacturing of primed mesenchymal stromal cells for therapy[J]. Nat Biomed Eng.

[65] Xu Y, Li-Ying Chan L, Chen S (2021). Optimization of UC-MSCs cold-chain storage by minimizing temperature fluctuations using an automatic cryopreservation system[J]. Cryobiology.

[66] Lechanteur C, Briquet A, Bettonville V (2021). MSC Manufacturing for Academic Clinical Trials: From a Clinical-Grade to a Full GMP-Compliant Process[J]. Cells.

[67] Strecanska M, Sekelova T, Smolinska V (2025). Automated Manufacturing Processes and Platforms for Large-scale Production of Clinical-grade Mesenchymal Stem/ Stromal Cells[J]. Stem Cell Rev Rep.

